# Forward entrainment: Psychophysics, neural correlates, and function

**DOI:** 10.3758/s13423-022-02220-y

**Published:** 2022-12-02

**Authors:** Kourosh Saberi, Gregory Hickok

**Affiliations:** 1 Department of Cognitive Sciences, University of California, Irvine, CA 92697 USA; 2 Department of Language Science, University of California, Irvine, CA USA

**Keywords:** Entrainment, Attention, Periodicity, Phase, Signal detection

## Abstract

We define forward entrainment as that part of behavioral or neural entrainment that outlasts the entraining stimulus. In this review, we examine conditions under which one may optimally observe forward entrainment. In Part 1, we review and evaluate studies that have observed forward entrainment using a variety of psychophysical methods (detection, discrimination, and reaction times), different target stimuli (tones, noise, and gaps), different entraining sequences (sinusoidal, rectangular, or sawtooth waveforms), a variety of physiological measures (MEG, EEG, ECoG, CSD), in different modalities (auditory and visual), across modalities (audiovisual and auditory-motor), and in different species. In Part 2, we describe those experimental conditions that place constraints on the magnitude of forward entrainment, including an evaluation of the effects of signal uncertainty and attention, temporal envelope complexity, signal-to-noise ratio (SNR), rhythmic rate, prior experience, and intersubject variability. In Part 3 we theorize on potential mechanisms and propose that forward entrainment may instantiate a dynamic auditory afterimage that lasts a fraction of a second to minimize prediction error in signal processing.

## Introduction

An extensive body of literature has investigated neural and psychophysical entrainment to periodic stimuli in different sensory modalities using a variety of experimental methods. These studies have shown that neural activity patterns at several levels of the cortical hierarchy phase lock to periodic stimuli and that cortical entrainment to the stimulus modulation envelope is both correlated with and predictive of behavioral measures. For reviews, see Sameiro-Barbosa and Geiser (2016), VanRullen (2016, 2018), Zoefel and VanRullen (2017), Haegens and Zion Golumbic (2018), Obleser and Kayser (2019), and Bauer et al. (2020). The current study focuses exclusively on a subset of these studies that have shown sustained entrainment (neural or psychophysical) *after* termination of the driving stimulus. We use the term “forward entrainment” to refer to that part of the entrainment process that outlasts the entraining stimulus, analogous to the concept of forward masking in psychoacoustics where masking effects are observed in signal detection after the masking sound has terminated. We contrast this to “simultaneous entrainment” that describes phenomena that are observed while the entraining stimulus is ongoing. We begin with an overview of studies that have shown forward entrainment (including our own work), we then describe constraints on experimental conditions that optimize detection of forward entrainment, and conclude with a discussion of how entrainment is evaluated across disciplines (physics, neurophysiology, cognitive science) and consider potential mechanisms that underlie forward entrainment.

## Evidence for entrainment

Forward entrainment typically lasts a fraction of a second and dissipates rapidly after the equivalent of three or four cycles of the entraining modulation envelope had it continued. In this section, we review those studies that have shown existence of such brief entrainment in different sensory modalities and using a variety of methodological approaches and measurement techniques. We intentionally exclude studies of informational or symbolic cuing (Correa et al., 2004; Coull & Nobre, 1998; Posner, 1980; Stefanics et al., 2010; Treisman, 1963; Xu et al., 2021) and focus instead on those that use implicit cues to capture attention or other involuntary rhythmic-coding (automatic) processes. These studies are summarized in Table 1.

**Table 1:** Selected studies that have demonstrated forward entrainment

Study	Paradigm	Entrained signal	Entraining sequence	Entraining frequency (Hz)
Lawrance et al. (2014)	Detection	Noise pulses	Noise pulses	4
Hickok et al. (2015)	Detection	Tone	AM noise	3
Farahbod et al. (2020)	Detection	Tone	AM noise	2, 3, 5
Forseth et al. (2020)*	Detection	Tone, ECoG	AM noise	3
Barnes and Jones (2000)	Discrimination	Silent gaps	Temporal intervals	1.7
Jones et al. (2002)	Discrimination	Tone	Tone sequence	1.7
de Graaf et al. (2013)*	Discrimination (visual)	x or +, MEG	Flickering annuli	5.3, 10.6
Spaak et al. (2014)*	Discrimination (visual)	Sinewave grating, MEG	Square flashes	10
van Bree et al. (2021)*	Identification	Words, MEG, EEG	tACS, Rhythmic Words	2, 3
Ellis and Jones (2010)	RT	Tone	Tone sequence	1, 2, 4
Lange (2009)*	RT	Gap, EEG	Tone sequence	1.8
Rimmele et al. (2011)*	RT	Tone, EEG	Tone sequence	5
Sanabria and Correa (2013)*	RT	Tone, EEG	Tone sequence	1.1, 2.5
Lakatos et al. (2013)	Neural	CSD, MUA	Tone sequence	0.8, 1.6, 3.2, 6.2
Simon and Wallace (2017)	Neural	EEG	AM noise	3

*Behavioral and neural

### Psychophysical detection and discrimination in forward entrainment

**Fig. 1:**
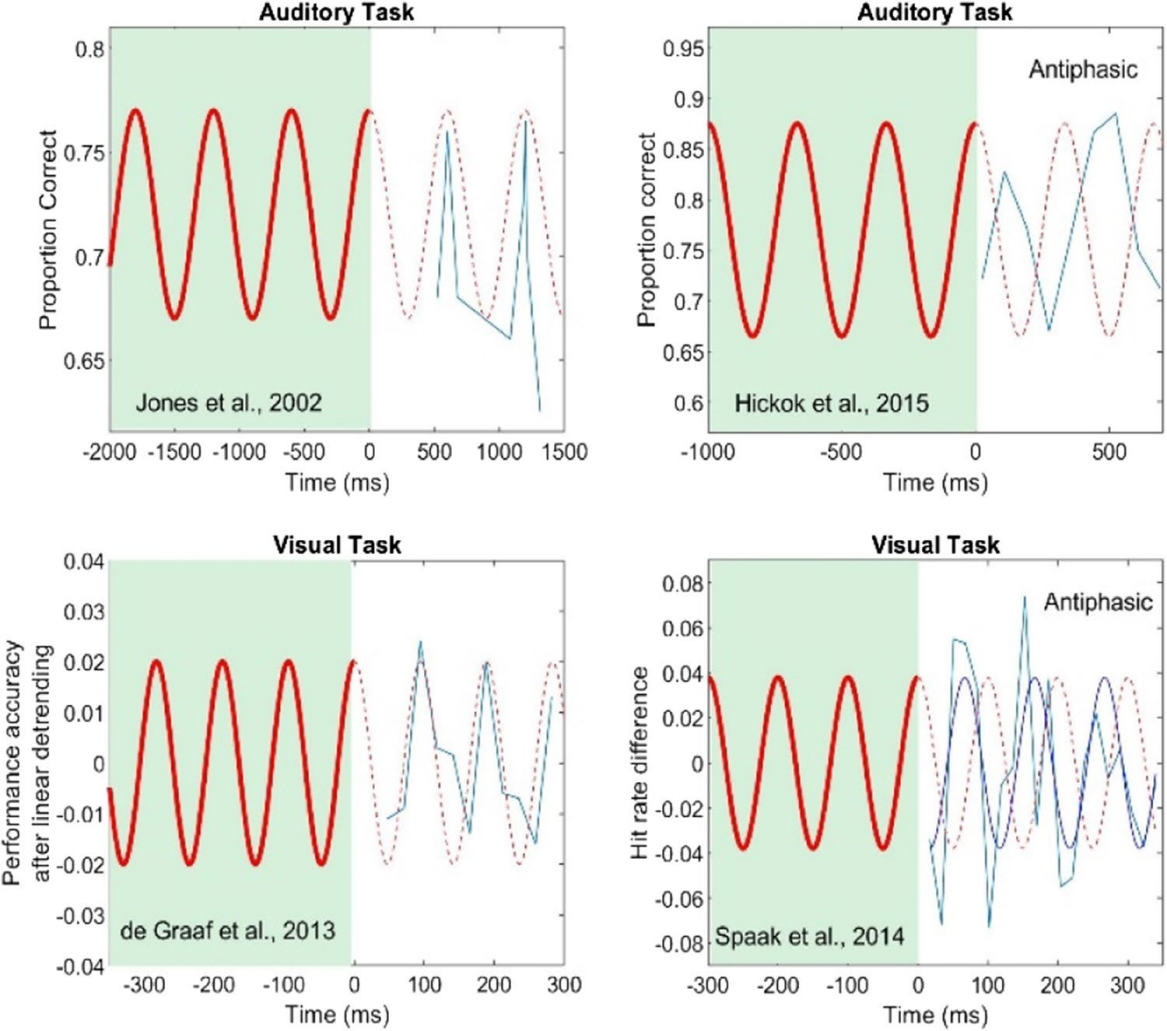
Results from four psychophysical studies that have shown multicycle forward entrainment. Top two panels show results from auditory tasks, and bottom two from vision tasks. Left panels show an in-phase pattern of forward entrainment and right panels show an antiphasic pattern

Figure 1 shows data from four different psychophysical studies that have demonstrated forward entrainment.^1^ The top two panels show results from auditory experiments and the bottom two from vision experiments. In each panel, the green-shaded region represents the period during which an entraining sequence was active and ongoing. Time zero represents the point at which the forward entrainment period begins. For clarity, we only show the last few cycles of the driving sequence. The red sinusoidal functions are a schematic representation of the frequency and phase of the driving modulator (entraining stimulus) and not the actual shape of the sequence envelopes used, which ranged from rectangular and sinusoidal acoustic envelopes to flickering annuli or square patches in visual tasks as described more fully below.

Figure 1A displays results from the seminal work of Jones et al. (2002). The results shown are concatenated from two experiments that separately investigated pitch discrimination at different temporal positions after termination of the entraining stimulus (Figs. 3 and 4 of Jones et al., 2002, from 23 subjects). The driving sequence was a binaural (diotic) set of nine tones with a fixed intertone interval of 600 ms. The first tone in the driving sequence was called the *standard* tone, the frequency of which was randomly selected from a closed set of five values and the duration of which was 150 ms. This was followed by eight tones (60 ms each) with random frequencies, for a total of nine tones in the driving sequence. After the final tone in the driving sequence, the *comparison* tone was presented. This comparison was 150 ms in duration and either had the same frequency (pitch) as the standard or was higher or lower by one semitone. The subject’s task was to indicate whether the pitch of the comparison tone was higher, lower, or the same as that of the standard. The critical variable of interest was the onset time of the comparison tone, which occurred either at the expected temporal interval (600 ms) or slightly off (one of four shifted onset times 524, 579, 621, and 676 ms). In a second experiment, the comparison tone was presented at 1,200 ms (twice the intertone interval of the driving sequence) to investigate the persistence of oscillatory effects in pitch discrimination. Their results clearly showed a cyclic pattern in pitch discrimination driven by the temporal expectancy set by the driving sequence. They speculated that this effect is based on attentional capture and a purely reflexive adaptive shift of attention in time toward the temporal locus of the target sound. Jones and colleagues have confirmed these general findings in several related or follow-up studies (Barnes & Jones, 2000; Barnes & Johnston, 2010; Ellis & Jones, 2010; Jones et al., 2006).

Figure 1B shows results of an auditory signal-detection study (Hickok et al., 2015). Here, the entraining stimulus was a 3-Hz sinusoidal amplitude modulated (SAM) noise that terminated on the cosine phase of the modulating envelope. The entraining stimulus was then followed immediately by steady-state (flat envelope) noise, the amplitude of which matched the peak of the modulating noise that preceded it. This allowed for a seamless transition between the modulating and steady-state noise segments without introduction of acoustic artifacts (see Fig. 1 of Hickok et al., 2015). The signal to be detected was a brief 50-ms tone pulse. On each trial, the tone was randomly presented during the steady-state noise at one of nine temporal positions, corresponding to two full cycles of the driving modulator had it continued (spaced evenly at 0.5π-radian or quarter-cycle intervals). On each trial, the tone’s intensity was randomly selected from one of five levels spanning a 12-dB range, sufficient to generate performance levels from near chance to near perfect detection. This level uncertainty appears to be important in observing forward entrainment in *near-threshold* signal detection (Farahbod et al., 2020).

Two findings from this study are immediately apparent. First, there is a cyclic pattern in signal detection that lasts for two cycles after termination of the driving modulator, consistent with findings on pitch discrimination by Jones et al. (2002). Second, and contrary to Jones et al., an antiphasic pattern in signal detection is observed with best performance near the temporal position at which the listener may have expected troughs of the driving modulator to occur (had the modulating entrainer continued), and worst performance at phases corresponding to where the listener expected peaks to occur. In most entrainment studies, the driving stimulus is of the same class as the signal to be detected, for example, tone sequence and tone signal. In the case of Hickok et al. (2015), the driving stimulus was noise, i.e., what is to be avoided. To optimize performance, listeners may have implicitly adopted a “listening-in-the-dip” strategy where signal-to-noise ratio (SNR) would be most favorable, a phenomenon well established in auditory psychophysics (Festen & Plomp, 1990; Hopkins & Moore, 2009; Peters et al., 1998). Listening in the dip allows subjects to take advantage of “glimpses” in the troughs of the expected modulating masker. In Jones et al. (2002) subjects heard the standard-comparison tones in quiet at suprathreshold levels where attending to the exact in-phase temporal positions would be beneficial (instead of focusing on the gaps between tones). In other words, in the Hickok et al. case, the noise is what the subjects were trying to avoid (to extract the tonal signal), hence an antiphasic pattern that entrains against the noise modulation, whereas in the Jones study, there is no noise to avoid, and hence no need to listen at the dips (gaps). This antiphasic pattern, coupled with findings on signal uncertainty (Farahbod et al., 2020) suggests that forward entrainment may be largely attention driven (even if implicitly so) rather than by a bottom-up neurophysiological mechanism. Findings from Hickok et al. (2015) have been supported by studies using nearly identical stimuli both psychophysically (Farahbod et al., 2020; Forseth et al., 2020; Henry et al., 2022; Saberi & Hickok, 2022a) and neurophysiologically (Forseth et al., 2020; Simon & Wallace, 2017).

The bottom panels of Fig. 1 show findings from two vision studies that have demonstrated forward entrainment in psychophysical signal detection. In the study by de Graaf et al. (2013), subjects were required to detect which of two visual targets (+ or x) briefly (11.8 ms) flashed on the screen. The entrainment sequence preceding the target comprised flashing annuli that flickered at one of two harmonically related frequencies (~5 or 10 Hz). They found a rhythmic pattern of behavioral performance that lasted for three cycles after termination of the entraining stimulus (Fig. 1C, reproduced from Fig. 3A of de Graaf et al., 2013). The oscillatory pattern of performance was restricted to 10 Hz, regardless of whether the entraining frequency was 5 or 10 Hz, consistent with MEG (magnetoencephalography) measures in the same 15 subjects (see Neurophysiological findings in forward entrainment section below). Figure 1D shows findings from Spaak et al. (2014) reproduced from their Fig. 1C. In the Spaak study, the entraining stimulus comprised brief (17-ms) flashes presented rhythmically at a rate of 10 Hz in one visual hemifield, and simultaneously, arrhythmically (jittered) at an *average* rate of 10 Hz to the contralateral hemifield. The near-threshold target was presented either in the hemifield that carried the rhythmic sequence or the hemifield that carried the arrhythmic (jittered) sequence. The subject’s task was to identify the hemifield within which the target appeared. On each trial the target was presented randomly at one of 20 discrete temporal positions *after* termination of the driving sequence. They found behavioral forward entrainment for three cycles after termination of the entraining stimulus. The blue sinusoid in Fig. 1D represents Spaak et al.’s best-fitting 10-Hz sinusoid to their data. Note that unlike de Graaf, they found that forward entrainment is antiphasic, similar to that reported by Hickok et al. (2015). The key similarity is that both studies have employed *near-threshold* signal intensities: in the Hickok et al. study, signal detection was limited by external noise, whereas in the Spaak study it was limited by internal noise (barely visible sine wave grating). Similar to de Graaf et al. (2013), the behavioral findings of Spaak et al. were consistent with MEG measurements of alpha cortical activity patterns in the same subjects, demonstrating forward neural entrainment that outlasted the entraining sequence for several cycles. The psychophysical results of de Graaf and Spaak are consistent with several other vision studies (Doherty et al., 2005; Mathewson, 2012).

Three other psychophysical studies are noteworthy, each providing a unique perspective into understanding the mechanisms of forward entrainment. The first is a noise-in-noise detection study by Lawrance et al. (2014), who investigated how a rhythmic noise sequence can preferentially affect the detection of a subsequent near-threshold noise signal. There are important parallels between this study and Hickok et al. (2015). First, the Lawrance study used signals whose intensity was near threshold. Second, the signal (noise) was to be detected in a continuous background noise after termination of the driving sequence. Third, the driving sequence itself was made up of amplitude-modulated noise. The entraining sound was a sequence of seven brief (25-ms) rectangular noise bursts superimposed on top of a continuous background noise and presented at a rate of 4 Hz. The intensity of each pulse in the entraining sequence was progressively decreased to generate the percept of a periodic sound that faded into the background noise. This section of the stimulus was followed immediately by a steady-state section that either contained or did not contain a signal to be detected. The signal comprised a set of five equal-amplitude noise bursts (25 ms each). On each trial of a two-interval forced-choice (2IFC) task, both a signal and a no-signal stimulus were presented in random order. The subject’s task was to determine which of the two intervals contained the target signal (i.e., the five noise bursts). There were two experimental conditions: (1) the *target* was rhythmic (4 Hz), and (2) the target was arrhythmic with random interburst intervals (2.9–6.7 Hz). Lawrance et al. found that 21 of 26 subjects showed an improvement in detection of rhythmic over arrhythmic targets following the termination of the entrainment sequence. The improvement averaged to approximately 1.5 dB SNR, though across individuals, this advantage could be as high as 3–5 dB. One difference between the results of Lawrance et al. (2014) and those of Hickok et al. (2015) is that the former found better performance for in-phase targets whereas the latter showed best performance for signals that were antiphasic to the entraining stimulus. Both studies used amplitude-modulating noise as the entraining stimulus, and both required subjects to detect a signal in a background of steady-state noise after termination of the entrainment segment. There is, however, a fundamental difference between the two studies in the nature of the target signal to be detected. In the Hickok study, the signal was a pure tone. As discussed above, an ideal observer would adopt a strategy to optimize performance by listening at the expected dips of the masker (had the masker modulation continued). This would generate an antiphasic pattern of performance, as observed. In the Lawrance study, however, the signal was a noise burst with spectrotemporal and statistical properties identical to those of the masker. An implicit strategy of listening in the modulating masker would simply result in “filling in” the gap with a statistically identical noise burst (signal), resulting in a flat noise envelope (and no signal to be detected). It would therefore be advantageous to listen for the noise signal at a point in time where it may be expected.

Another study we’d like to highlight in this section is that by Barnes and Jones (2000). It is an important study in that it observes forward entrainment using an interesting and categorically different type of discrimination task. Barnes and Jones measured the ability of listeners to determine if two temporal intervals (silent gaps) whose edges were marked by brief (60-ms) tones were the same or different. Each temporal interval of the entraining sequence was 600 ms, i.e., a silent temporal interval bounded by short tone pulses. The last temporal interval of the entraining sequence, however, was selected from one of five equally likely values centered on 600 ms (ranging from 524 to 676 ms). This final “silent” interval was called the *standard* to which a *comparison* “silent” interval was to be compared. Two interesting findings emerged. First, they found that performance was better by approximately 20% when the entraining intervals matched the standard, compared to when no entraining sequence was present. This advantage declined from 20% to 10% when the standard was slightly off (by about 20 ms) relative to the entraining sequence intervals, and to near 0% advantage when the standard and entraining intervals were significantly different (~75 ms). In a very interesting follow-up experiment, they investigated the nature of the internal temporal referent generated by the entraining sequence. The goal was to determine if this referent was based on stored central memories of temporal intervals (i.e., a cognitive effect) or a consequence of an implicit oscillatory resonance. They found that a harmonically related entraining interval (300 ms) more effectively entrained the 600-ms standard, than an entraining interval (500 ms) that was closer in duration (but inharmonically related) to the 600-ms standard. This finding supported an oscillatory model of forward entrainment as contrasted to a central memory-storage model.

Finally, in a recent paper, van Bree et al. (2021) used speech stimuli to show that forward entrainment is not a purely bottom-up process but also has a predictive higher-order component. They rhythmically stimulated the cortex using transcranial alternating current stimulation (tACS) at 3 Hz (note that the stimulus is not auditory). They measured the ability of listeners to identify a monosyllabic target word presented in noise at one of six delays after termination of the tACS entrainer. The six delays covered a single cycle of a 3-Hz modulating envelope. They found that word-identification accuracy depended, in a modulatory manner, on the delay between the end of the tACS entrainer and the perceptual center of the target word (Morton et al., 1976). They further reported a neural correlate of this behavioral modulation. Using MEG and EEG (electroencephalography), they found that a rhythmic sequence of intelligible words presented at a rate of either 2 or 3 Hz produced forward entrainment of neural activity in the parietal cortex. Importantly, they were able to predict results of their behavioral measurements (accuracy) from neural oscillatory patterns. Specifically, they predicted the optimum phase delay (between rhythmic intelligible speech and EEG responses) that generated the highest accuracy in word identification. The sustained oscillatory pattern in neural activity was not observed for unintelligible words consisting of single-channel vocoded words that were effectively perceived as sequences of noise bursts. Prior studies have shown neural forward entrainment for basic auditory stimuli. Why, then, was neural forward entrainment not observed for unintelligible word sequences in the van Bree study? They suggest that predictive oscillatory mechanisms may possibly be stronger for intelligible speech, whereas noise-like unintelligible word sequences that have less real-world predictive relevance may just generate a weaker (and shorter) sustained oscillatory neural response. We suggest that another potential explanation is that perhaps intelligible words simply activate implicit attentional networks more strongly than unintelligible words, and as Lakatos et al. (2013) have demonstrated, neural forward entrainment in the monkey auditory cortex requires attention and disappears when the animal is inattentive (see section on Neurophysiological findings in forward entrainment).  Farahbod et al. (2020) and Saberi and Hickok (2022a) have also argued for a critical role of attentional capture in forward entrainment.

### Reaction-time paradigms in forward entrainment

In the previous section we examined forward entrainment in psychophysical detection and discrimination paradigms in which performance was evaluated using an accuracy measure (e.g., proportion correct). In this section, we review studies of forward entrainment that have employed reaction time (RT) as a dependent measure. While RT and accuracy are often correlated (e.g., speed-accuracy trade-off), they are not two sides of the same coin (Kahana & Loftus, 1999; Prinzmetal et al., 2005). One measures the amount of information that a sensory or cognitive process contains (accuracy), and the other the time it takes to complete the process (RT). Some studies have shown that a variable can have a significant impact on performance accuracy without affecting RTs (MacLeod & Nelson, 1984; Sternberg, 1969), and others have shown significant effects on RT without a correlated change in accuracy that is far from ceiling (Kahana & Loftus, 1999; Sanders et al., 1974; Santee & Egeth, 1982). It is therefore important to explore both accuracy and RT measures in developing theories of psychophysical entrainment. We describe here four auditory psychophysical studies (using four different tasks) that have demonstrated auditory forward entrainment in an RT paradigm.

**Fig. 2:**
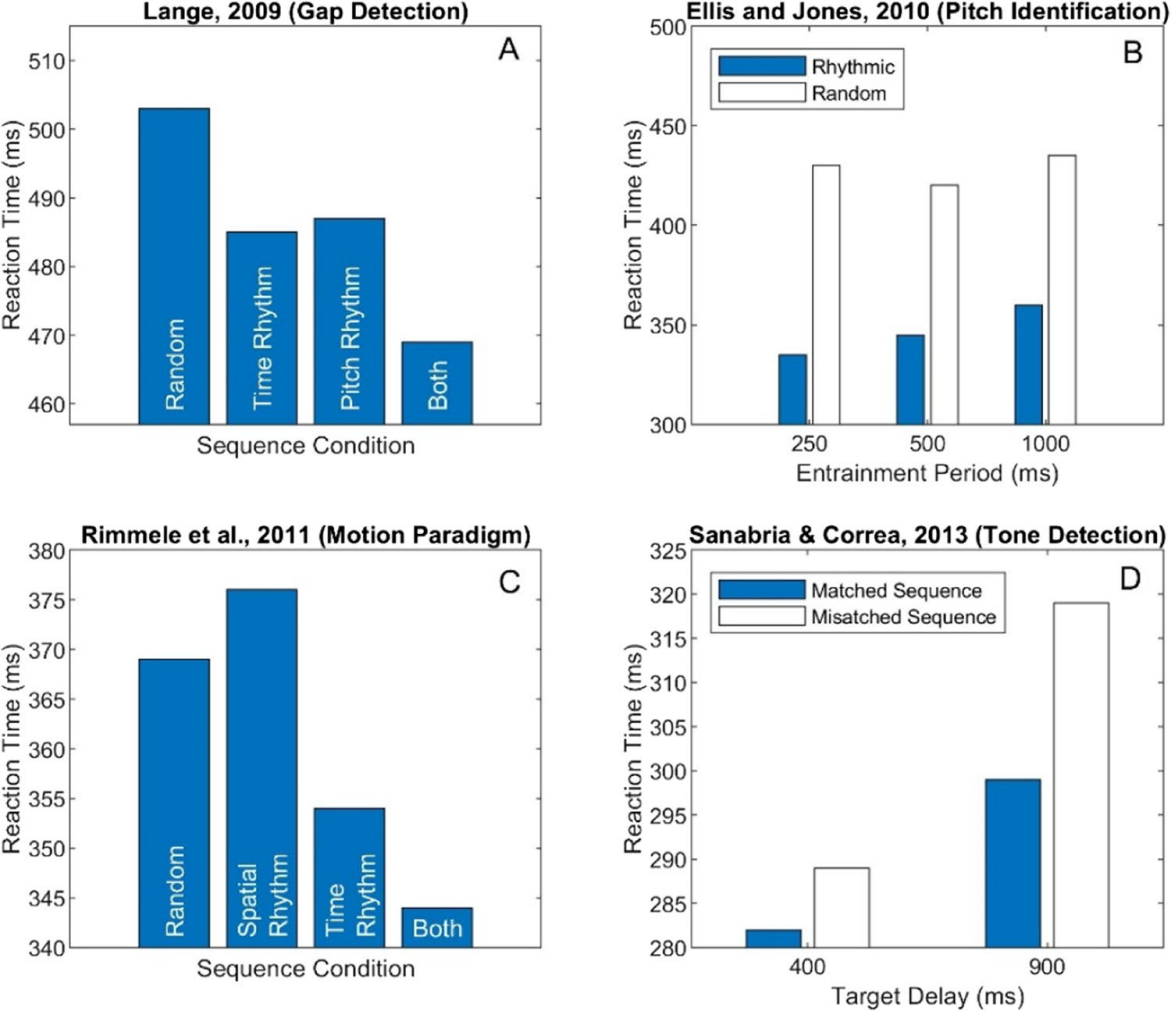
Results from four studies that have shown forward entrainment using reaction-time (RT) measures in four different auditory tasks (gap detection, pitch identification, motion paradigm, and tone detection). See text for a detailed explanation of each study

Lange (2009) has shown that RTs for detection of a brief gap (10 ms) in the middle of a 100-ms target sound can be significantly faster when the target is preceded by an entraining sequence presented at a rate of ~2 Hz. The task was to respond as fast as possible in a 2IFC task (is there a gap present or not?). The entrainment sequence implicitly cued one of three things about the target: (1) when the target may occur, (2) its pitch (via an ascending pitch sequence), or (3) both time *and* pitch. Accuracy (detection performance) was intentionally set to a high level in all cases (average 94%) so that the effects of forward entrainment on RT could be isolated. Results are shown in Fig. 2A. Compared to a control sequence (randomized time/pitch), RTs were significantly faster when either the target’s time or pitch were independently cued. RTs were fastest when the entraining sequence cued *both* the time of occurrence and pitch of the target sound within which the gap occurred. Lange (2009) also reported a neural correlate of these psychophysical findings in both the N1 and P300 components of the ERP (evoked response potential) signal generated by the rhythmic sequence compared to the arrhythmic (random) sequence.

Ellis and Jones (2010) designed an interesting entrainment sequence that simultaneously cued for three harmonically related entrainment periods: 250, 500, and 1,000 ms (4, 2, and 1 Hz). These periods were interleaved within the same entraining sequence, creating a nested hierarchical structure of multiple entrainment periods. Each sequence comprised brief tone pulses followed by a target tone at *one* of the three cued periods after termination of the entraining sequence. All tones within a sequence had a fixed frequency. The target tone had a different frequency that was either higher or lower than that of the entraining tones. The subject’s task was to determine if the pitch of the target was high or low (a pitch-identification task). Subjects had no difficulty with this task as evidenced from high accuracy rates (~98%). Figure 2B shows their results reproduced from Fig. 5A of their study. RTs for rhythmic sequences were significantly faster than those for scrambled arrhythmic sequences at all three entrainment periods, even though these three entraining periods were interleaved within the same sequence.

Rimmele et al. (2011) investigated both RTs and detection sensitivity (*d’* ) in a study that combined temporal and spatial (location) regularity by use of auditory motion stimuli. Four possible entraining sequence permutations included motion stimuli that had temporal regularity only (rhythmic sequence), spatial regularity only (a sequence of 14 distinct spatial locations), temporal *and* spatial regularity, or no regularity (random temporal phase and spatial location). At the end of the entrainment sequence, a brief (occluding) noise was presented, followed by either a target signal (tone) or no signal. The subject’s task was to respond as fast as possible if they detected a signal (go/no-go task). Figure 2C shows their results. They found faster RTs (by about 25 ms) and slightly higher *d’*s for targets preceded by a temporal entrainment sequence but not for targets preceded by spatial regularity. Their results were consistent with ERP measures where the P1, N1, and N2 components (reflecting pre-motor response, early perceptual processing, and task-related responses) were modulated by the rhythmic entrainment cues, but not by spatial entrainment.

Sanabria and Correa (2013) reported similar findings in a tone-detection task. They showed that when a target tone is preceded by a rhythmic sequence of tones, RTs are faster when the target inter-stimulus intervals (ISIs) matched the rhythmic cadence of the entraining sequence. This behavioral improvement in RT was accompanied by correlated modulations of the N1 and P2 potentials of ERP recordings in the same subjects. Interestingly, there was a significant difference in the N1 component in response to a target ISI for which a statistically significant behavioral improvement had not been observed. This suggests that the underlying neural process may not have triggered a sufficiently strong behavioral response to be observed in psychophysical measurements.

One interesting observation about these four RT studies is the absence of an antiphasic pattern of performance. Improvements in RT occur when the signal is in-phase with the temporal expectancies set by the entraining sequence. RT measures, however, are made in quiet at suprathreshold signal intensities (near ceiling) with a sequence that is of the same class as the signal type (tones). No prior RT study has used near-threshold signals in noise where avoiding the temporal expectancies set by the peak of the noise modulator would be the optimum strategy in isolating the signal.

What do RT studies of forward entrainment tell us beyond findings from detection and discrimination paradigms? There is clearly a correlation between the two classes of studies in that entraining sequences can improve accuracy (amount of information) and reduce RTs (time it takes to accumulate sufficient information for a decision). The magnitude of improvements in RT resulting from forward entrainment is generally under 100 ms (Ellis & Jones, 2010) and more often under 40 ms (Lange, 2009; Rimmele et al., 2011; Sanabria & Correa, 2013). The magnitude of improvements in performance accuracy for entrained auditory signals is typically between 10% and 20% (Farahbod et al., 2020; Henry et al., 2022; Hickok et al., 2015; Jones et al., 2002). How do we compare these two measures? There have been a number of attempts to relate RT to accuracy using detection-theoretic modeling approaches (Kahana & Loftus, 1999; Kornblum, 1973; Laming, 1968, 1986; Ratcliff, 1978, 2018; Wagenmakers, 2007). These, however, require RT measurements at several different SNRs (or detection levels). Currently, no study has measured entrainment effects simultaneously on RT and accuracy at different performance levels. Typically, studies using RT as a dependent measure maintain accuracy performance at a single value near ceiling level. It would be useful to investigate forward entrainment using joint RT and accuracy measures, as it is likely that these two measures are evaluating complementary processes from converging perspectives.

### Neurophysiological findings in forward entrainment

Several studies have observed neural forward entrainment using a variety of measurement techniques from ECoG, EEG, and MEG in humans to single or multiunit electrode recordings in animals. Some of these studies have concurrently measured psychophysical performance that appears correlated with the modulating neural activity patterns. In this section we focus on four of these studies, a recent ECoG study of awake human subjects performing an auditory signal-detection task while their brain activity was directly recorded using intracranial (depth and/or grid) electrodes (Forseth et al., 2020), an EEG study showing antiphasic auditory forward entrainment in fronto-central brain regions while attending to post-stimulus auditory targets but not when attending to audiovisual targets (Simon & Wallace, 2017), an MEG study that reported forward entrainment of neural activity in occipito-posterior areas of the cortex in response to rhythmic visual stimuli (Spaak, 2014), and an animal neurophysiological study that used microelectrodes to directly record from the awake monkey primary auditory cortex and found forward entrainment that was critically dependent on attention to the sound sequence (Lakatos et al., 2013). Two of these studies (Forseth et al., 2020; Simon & Wallace, 2017) employed stimuli that were identical to those used by Hickok et al. (2015). We describe each of these studies in more detail below.

**Fig. 3:**
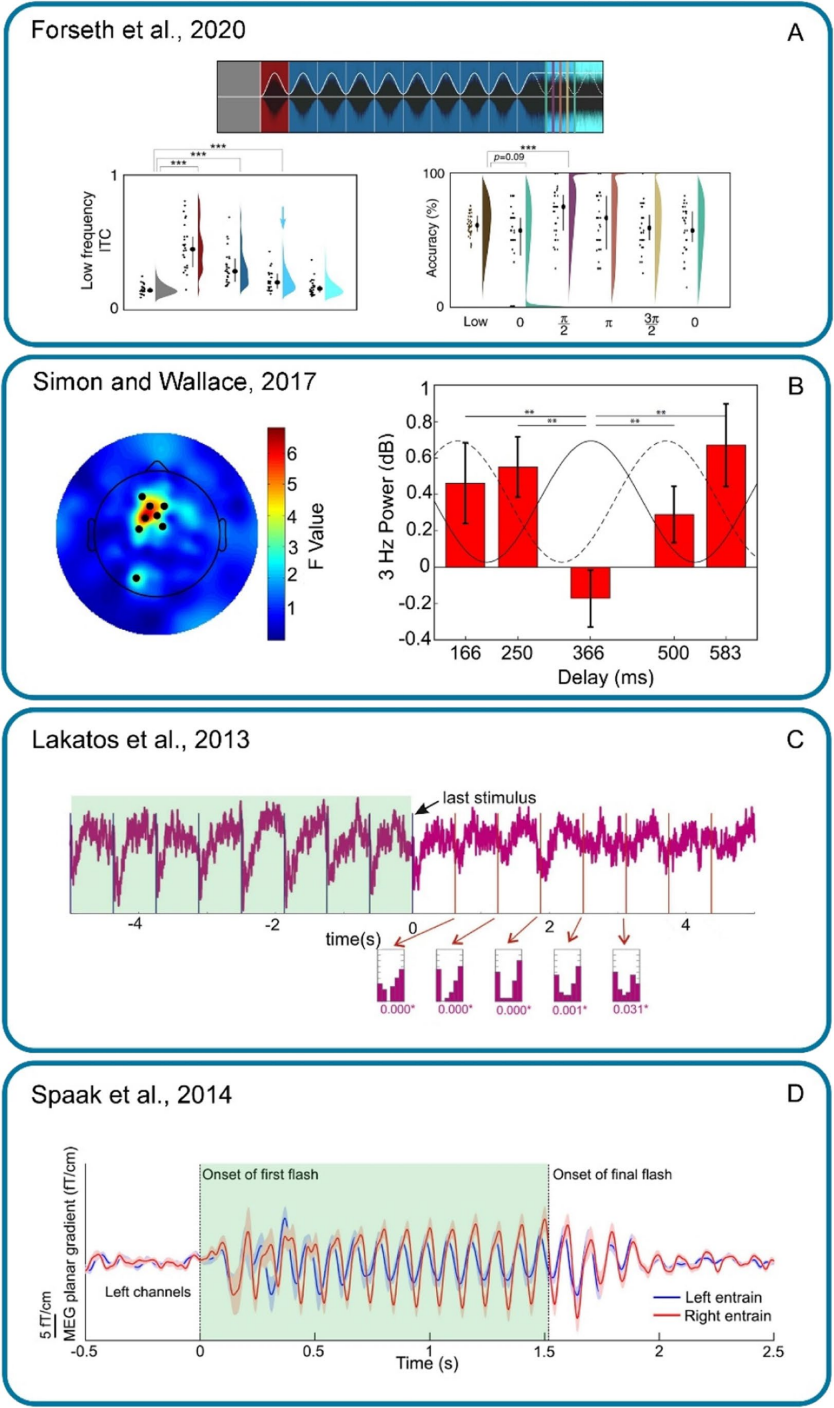
Four neurophysiological studies of forward entrainment using four different measurement methods (ECoG, EEG, CSD, and MEG). See text for details. Permission to use granted by Elsevier under STM (The International Association of Scientific, Technical and Medical Publishers) permission guidelines, and by The Journal of Neuroscience and Nature Communications under the terms of the Creative Commons Attribution 4.0 International License

Forseth et al. (2020) measured oscillatory entrainment of cortical activity using ECoG (electrocorticography) in 37 patients. The patients were surgically fitted with depth or surface-grid electrodes (or both) and cortical recordings were made while the patient was awake and performing the same signal-detection task used by Hickok et al. in which an entraining modulating noise (3 Hz) was followed by steady-state (flat envelope) noise during which subjects were to detect a brief pure-tone signal. Figure 3A shows their results reproduced from their Figs. 4A, C, and D. Two of their findings are especially relevant here. First, they found that modulation of neural activity in the early auditory cortex (Heschl’s gyrus (HG) and the transverse temporal sulcus (TTS)) continues phase-locked to the driving modulator for one cycle after termination of stimulus modulation (mid-blue color in bottom-left panel of Fig. 3A marked by arrow). Second, they found that modulation of behavioral performance measured simultaneously during neural recordings outlasted the rhythmic stimulus for one cycle after termination of stimulus modulation (they only examined one cycle post modulation). Psychophysical results are shown in the bottom-right panel of Fig. 3A where performance at π/2 is significantly higher than baseline. These findings are consistent with Hickok et al. (2015) and Farahbod et al. (2020), though the exact phase at which best performance is observed is slightly different (by a quarter of a cycle).

**Fig. 4:**
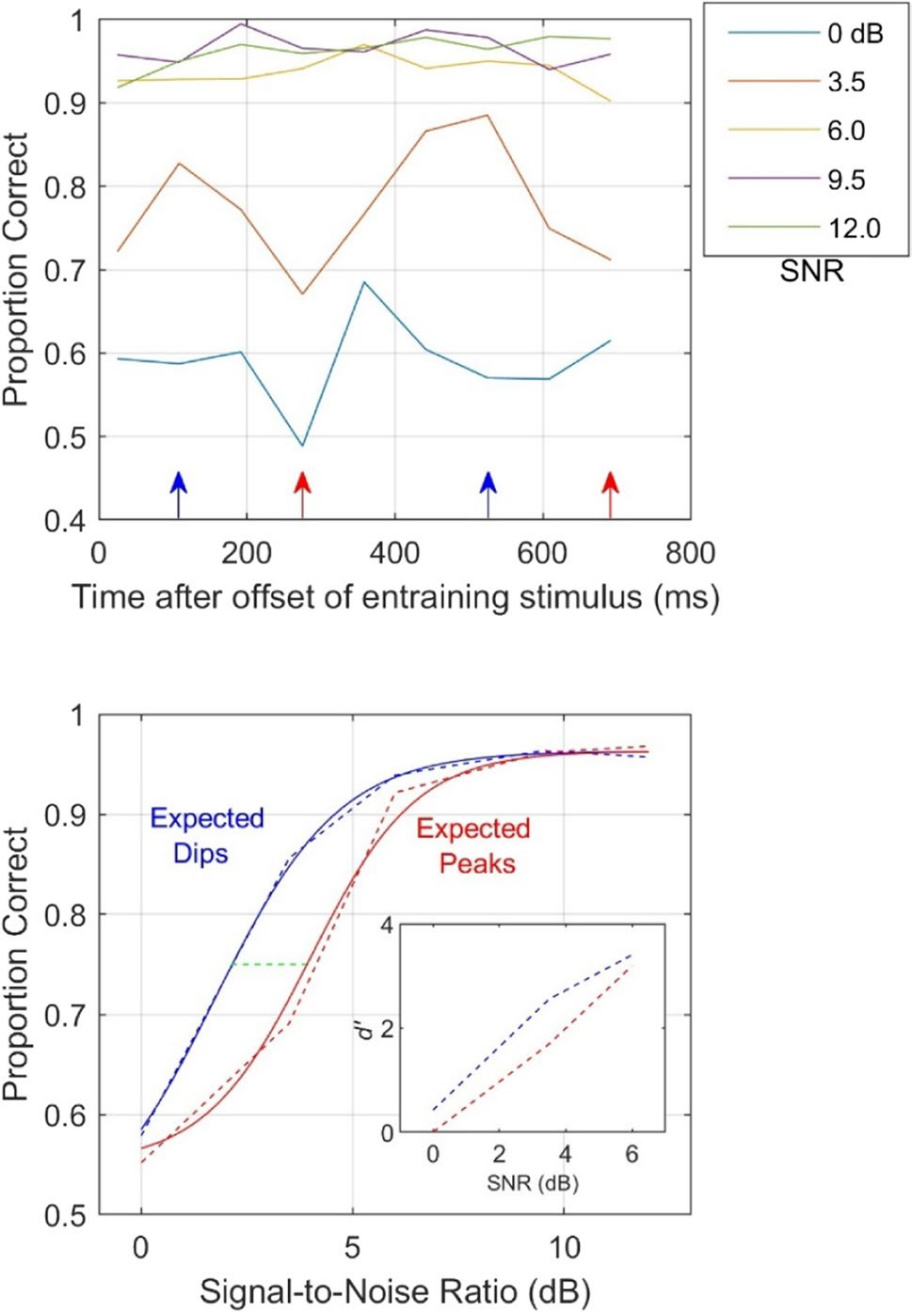
Top: Performance as a function of signal temporal position averaged across subjects. Time zero represents the end of the entraining stimulus. The parameter is signal-to-noise ratio (SNR), with the 3.5-dB condition showing data reported by Hickok et al. (2015). Bottom: Psychometric functions estimated from data in the top panel (arrows). Red and blue curves show functions generate from data marked by arrows of the same colors in the top panel. These arrows designate the peaks and dips of the curve at 3.5 dB in the top panel (orange), which is antiphasic to dips and peaks of the stimulus modulation envelope (had it continued). The blue curve is associated with the expected dips in the AM noise stimulus, and the red curve with the expected peaks. See text for details

Simon and Wallace (2017) also used stimuli identical to those used by Hickok et al. (2015) with the exception that after the entraining 3-Hz modulated noise terminated, the tone pulse was presented in *quiet* to isolate EEG measurements that exclusively reflected forward entrainment. The target tone was presented at one of five temporal positions after the end of the modulating noise. On a small proportion of trials, the target tone had a “deviant” frequency and the subject’s task was to press a key when the deviant tone was detected (oddball task) and do nothing otherwise. The deviant tone always occurred at a fixed delay (the third temporal position). The main findings from Simon and Wallace (2017) are shown in Fig. 3B (reproduced from their Fig. 6D). Time zero represents the end of the entraining stimulus, and the abscissa represents the delay between the end of the entrainment and the target tone. The solid black curve indicates the phase of the entraining stimulus (had it continued), and the dotted curve indicates what they refer to as brain phase (stimulus phase + a brain lag of 120 ms). They found that the magnitude of the 3-Hz power (the entraining frequency) showed significant phase dependency that was consistent with a 3-Hz oscillatory cycle. The power modulation at the fronto-central sites was antiphasic to the terminated driving modulator, similar to that reported for behavioral measurements by Hickok et al. (2015). This means that the forward oscillations induced by the entraining noise (after it had terminated) was substantially attenuated when the target pulse coincided with when the rhythmic noise would have peaked (had it continued). They speculated that “when the entrainment stimulus is irrelevant noise, the brain entrains *against* the noise and thus aims to enhance the processing of salient events happening during the gaps”, i.e., a listening-in-the-dips strategy.

Lakatos et al. (2013) measured current source density (CSD) and multiunit activity (MUA) from the primary auditory cortex (A1) of awake macaque monkeys as they were attending to sequences of pure-tone pulses presented at low rates (from 1.6 to 12.2 Hz). The monkeys were trained to respond to deviant-frequency tones in an oddball design. They found that *after* the termination of the driving sequence, neural activity continued to oscillate rhythmically (and in phase) with the discontinued driving sequence. This effect was critically dependent on attention and was absent for non-attended sequences. Figure 3C (reproduced from Fig. 3A of Lakatos et al. 2013) shows 10 s of averaged CSD activity in response to the 1.6-Hz tone sequence (5 s of stimulation and 5 s after the end of the entraining tone sequence). Time zero represents the end of the entraining stimulus (shaded green rectangle). The blue drop lines represent times at which the tone pulses occurred, and the red drop lines show time points at which these pulses would have occurred if the entraining stimulus had continued (note that negative CSD designates high excitability). The histograms below the CSD trace show the distribution of phases at these time points. Below the histograms, significant *p* values are displayed for each phase distribution. The sustained oscillating neural activity (forward entrainment) was observed for rhythm rates from 0.8 to 6.2 Hz, but not for the higher rate of 12.2 Hz. The range of rates for which neural forward entrainment was observed is approximately the same as those for which Farahbod et al. (2020) have reported behavioral forward entrainment, i.e., 2–5 Hz but not at higher rates.

Finally, forward neural entrainment has also been shown in other sensory domains. We highlight one of them here. Spaak et al. (2014) used an entraining stimulus comprised of a rhythmic sequence of visual flashes (10 Hz) while recording MEG signals from sensors overlying the occipito-posterior areas of the cortex. Figure 3D shows an example recording. The red and blue curves correspond to rhythmic stimulation in the right and left visual fields, respectively. The green-shaded region (added by us) shows the time period during which the visual flash sequence was presented. Note that after termination of the entrainment sequence, the cyclic MEG activity persists for several cycles. One interesting difference between this study in the visual modality and the auditory neurophysiological study by Lakatos is worth mentioning. Lakatos et al. suggest that the observed ongoing oscillatory neural activity is critically dependent on attention, and not observed when the animal is not attending to the target sequence. Spaak et al., however, suggest that the ongoing oscillatory pattern of neural (and behavioral) activity they have observed is likely a low-level process that taps into the kinetics of the neural system (similar to the resonance of a system) and not a process that has necessarily evolved to extract temporal information. As evidence for this interpretation, they note that the oscillatory pattern of behavioral performance they observed is antiphasic to the driving entrainment stimulus (see Psychophysical detection and discrimination, above). They argue that temporal expectancy would predict the opposite pattern and an enhancement of performance at in-phase delays. However, auditory psychophysical experiments that have investigated the role of stimulus uncertainty (Farahbod et al., 2020) suggest that the antiphasic pattern of behavioral performance is, in fact, consistent with an attentional process that promotes a “listening-in-the-dip” strategy. We should note that Spaak et al. (2014) do not dismiss the role of selective attention given the substantial evidence showing that neural oscillations are strongly affected by top-down attentional control (Bonnefond & Jensen, 2012; Haegens et al., 2011; Händel et al., 2011) and in fact suggest that it would be of significant interest to investigate how attention may interact with entrained oscillations they have observed in the visual cortex.

In addition to the four studies using four different neurophysiological methods highlighted here, there are a number of other similar studies in the auditory and visual domains that have demonstrated forward entrainment of neural activity in response to rhythmic stimuli (de Graaf, 2013; Kösem et al., 2018; Lange, 2009; Rimmele et al., 2011; Sanabria & Correa, 2013; Schmidt-Kassow et al., 2009; van Bree et al., 2021). Most of these have also simultaneously shown behavioral correlates that appear phase-locked to the terminated rhythmic stimulus. In summary, forward neural entrainment has been shown using a variety of recording methods (EEG, MEG, MUA, CSD, and ECoG) in humans and animals at multiple levels of the cortex, and across sensory modalities (auditory and visual domains).

## Constraints on forward entrainment

Forward entrainment is observed under some experimental conditions (as described in *Psychophysical detection and discrimination* above) and absent under other conditions. In this section, we describe those stimulus and experimental design conditions that place constraints on the magnitude of forward entrainment.

### Effects of signal uncertainty

Selective attention has been shown to improve psychophysical performance under conditions of uncertainly in a number of standard auditory tasks, for example in tasks involving the detection of tones of uncertain frequency (Dai et al., 1991; Hafter & Saberi, 2001; Hafter et al., 2008; Schlauch & Hafter, 1991; Wright & Fitzgerald, 2017), uncertain duration (Dai & Wright, 1995), or uncertain time of occurrence (Bourbon et al., 1966). As uncertainty increases and predictability decreases, the system’s limited attentional resources are allocated to monitoring specific time points set by the rhythmic entraining sequence, resulting in a brief attentional cadence after termination of the entraining stimulus. Removal of signal uncertainty mitigates the need for selective attention and diminishes potential post-stimulus modulatory effects in signal detection. This is what Farahbod et al. (2020) observed when they removed level uncertainty in a forward entrainment paradigm. When signal levels and temporal positions of the signal were mixed within a block of trial, they observed forward entrainment in an antiphasic pattern, yet when they removed level uncertainty for the same listeners, no modulatory effect (no forward entrainment) was observed. Other studies of forward entrainment have also typically used a design in which some aspect of the stimulus is uncertain, usually by mixing signals of various delays or frequencies across trials within the same run (Barnes & Jones, 2000; Ellis & Jones, 2010; Forseth et al., 2020; Jones et al., 2002; Lange, 2009; Lawrance et al., 2014). At least in one case of failure to observe forward entrainment, a block design with no uncertainty in rhythmic target conditions was used (Lin et al., 2021; see also Saberi & Hickok, 2022b). This, however, is not always the case (Bauer et al., 2015), and additional studies on the role of uncertainty on forward entrainment are warranted.

### Effects of temporal envelope complexity

Nearly all studies of forward entrainment have employed simple rhythmic modulation patterns such as sinusoidal, squarewave, rectangular, or triangular sequences with a simple rhythm. To determine if the effects of masker modulation on signal detection can persist for more complex modulation patterns, Saberi and Hickok (2021) repeated the Hickok et al. (2015) study using noise maskers that were simultaneously modulated at more than one rate. Two such complex patterns were examined: (1) combined modulation rates of 2 and 3 Hz (i.e., the two rates at which the strongest forward entrainment was reported by Farahbod et al., 2020), and (2) combined modulation rates of 3 and 5 Hz. They found that performance did not follow the more complex shape of the modulating envelopes for combined rates, as it had for simple sinusoidal amplitude modulation at a single rate. This suggests that either the modulation pattern is too complex to affect signal detection or the combined frequencies result in an average modulation rate that is too high to yield entrainment (Farahbod et al., 2020; Saberi & Hickok, 2021).

### Effects of signal-to-noise ratio (SNR)

The top panel of Fig. 4 shows signal-detection performance in a forward entrainment paradigm at five SNRs ranging approximately 12 dB (Hickok et al., 2015; Saberi & Hickok, 2021). Time zero represents the end of the entraining stimulus. The parameter is SNR, with the 3.5-dB condition representing the data reported by Hickok et al. (2015), and the other four SNRs showing previously unpublished data from the same experiment. The 0-dB SNR represents baseline (lowest signal level tested). No modulatory effect is observed for two of the three highest SNRs, with only a mild bicyclic effect at SNR of 9.5 dB. While the absence or weak modulation effects at high SNRs is partially associated with ceiling effects, it may also be related to the fact that the higher SNRs reduce uncertainty and the need for selective attention. At the lowest SNR (0 dB) there does seem to be a dip in line with that seen for the 3.5-dB SNR data of Hickok et al. (2015).

The bottom panel of Fig. 4 shows a different approach to analyzing forward entrainment using the entire psychometric function. It shows performance as a function of SNR under two conditions. The two conditions are associated with the peaks and troughs of the 3.5-dB SNR function shown in the top panel (orange curve) where the largest modulation in performance was observed. In order to maximize the number of trials that generated each of two psychometric functions, the data were pooled across the two peaks (marked by blue arrows in the top panel) producing the blue psychometric function in the bottom panel, and the two dips (red arrows) producing the red psychometric function. The dashed lines in the bottom panel represent the actual data and the solid curves are modified logistic psychometric functions fitted to the data. These two psychometric functions use a much larger dataset at five SNRs to show differences in performance associated with the expected dips and peaks in the modulation waveform had it continued. For all SNRs, except for the near-ceiling SNR of 12 dB, performance is better for the blue function (associated with expected dips) than the red function (expected peaks). Note that these are antiphasic to the *performance* curves where the blue curve is associated with the peaks of the orange curve in the top panel (arrows), and the red curves with the dips. Except for the 3.5-dB SNR, the difference in performance at the other SNRs is notably small, in the order of 2–3% (compared to an approximately 17% difference at the 3.5-dB SNR). Nonetheless, their direction is consistent with that observed at the 3.5-dB SNR. The inset shows the same data plotted as 3-point psychometric functions in *d’* units derived from hit- and false-alarm rates. Threshold improvements (difference between blue and red curves) measured at the 75% performance level (green dashed line) is approximately 1.8 dB. This gain in performance, which is estimated from the entire psychometric function, is consistent with the 1.5-dB improvement reported by Lawrance et al. (2014), who also used a signal-in-noise paradigm to compare thresholds for a signal that was either in-phase (rhythmic) or random (arrhythmic) with respect to a terminated rhythmic noise sequence.

### Effects of experience and intersubject variability

In our recent work we have found what seems to be differences between experienced and inexperienced subjects that manifest largely in intersubject variability and the SNR at which strongest entrainment effects are observed (Saberi & Hickok, 2021). Similar intersubject variability has also been reported by Jones et al. (2002), Lawrence et al. (2014), Bauer et al. (2015), and Sun et al. (2021), where a proportion of their subjects show forward entrainment and a proportion do not under the same experimental conditions (see also Saberi & Hickok, 2022a). These proportions vary widely across studies. Further evidence of intersubject variability is that in cases where bicyclic patterns are observed, the phases at which a dip (or peak) in behavioral performance is seen do not always precisely line up across subjects. This is not that surprising given the statistical nature of performance and limited sample size, but is important because minor phase misalignments can diminish or flatten modulation patterns in behavioral performance when data are averaged across subjects (Saberi and Hickok, 2021). Subject-specific phase dependency has been reported for simultaneous entrainment (Henry & Obleser 2012), and a phase drift in the dips and peaks of performance has been reported by Farahbod et al. (2020) in a forward entrainment task as a function of the entraining modulation rate. Variable starting-phase effects have also been reported by Sun et al. (2021) for a proportion (35%) of their subjects who showed forward entrainment (see also a critique of Sun et al.’s findings by Saberi & Hickok, 2022a).

### Effects of rhythmic rate

Finally, forward entrainment is rate limited and lowpass in nature. Farahbod et al. (2020) tested auditory psychophysical entrainment for rhythmic rates of 2–32 Hz and found that it was strongest for rates of 2 or 3 Hz, weaker at 5 Hz, and nonexistent for rates from 8 to 32 Hz. The absence of post-stimulus entrainment at higher rates has also been observed in neural recordings. Lakatos et al. (2013) reported that neural activity continued to oscillate rhythmically after termination of an auditory entraining sequence (see Neurophysiological findings above) but only for rhythmic rates from 0.8 to 6.2 Hz, and not at the higher rate of 12.2 Hz. This range of rates is also in general agreement with, but somewhat lower than, those reported for temporal modulation transfer functions (TMTFs), which measure modulation detection thresholds as a function of modulation rate (Eddins, 1999; Scott & Humes, 1990) as well as with the firing-rate limits of auditory cortical neuron in response to AM sounds (Barton et al., 2012; Joris et al., 2004).

## Mechanisms

### What is entrainment?

Entrainment in physical systems occurs when the temporal dynamics of one system are captured by another, resulting in correlated activity beyond chance correlation. More restrictive definitions have been advanced by Haegens and Zion Golumbic (2018) and Obleser and Kayser (2019) that the entrained system be endogenously oscillating at a characteristic frequency (i.e., show natural self-sustained periodicity) and that the entraining system itself be an autonomous oscillator (see also Wilsch et al., 2020). This “coupled oscillators” definition, however, describes only a subclass of entrainment phenomena. In applied physics, an entrained system need not be endogenously periodic but can be in a default aperiodic or rest state; similarly, in the case of neural systems, a network can display scale-free or other non-oscillatory activity *prior* to entrainment (He, 2014; Maniscalco et al., 2018). Some have suggested using the term “neural tracking” or “envelope locking” for this set of phenomena. However, this distinction, while useful (and valid) for explaining certain neural phenomena using certain measurement methods, unnecessarily constrains the definition and fails to conform to broader classifications in physics. The more universal classification scheme includes the entrainment of intrinsically non-oscillatory networks whose computations are often at scales too fine-grained for (and opaque to) extracranial recordings.^2^ Other non-oscillatory entrainment phenomena include stochastic (or noise-induced) entrainment that enhances a system’s nonlinear response to weak or subthreshold signals (Collins et al., 1996; Mori & Kai, 2002; Read & Siegel, 1996; Wang & Peskin, 2015), aperiodic entrainment that allows irregular neural activity to reliably transmit critical information about external nonperiodic sensory events (Butzin et al., 2015; Mainen & Sejnowski, 1995; Phogat & Parmananda, 2018), chaotic synchronization where systems with close initial conditions in phase space desynchronize and then, counterintuitively, converge via entrainment, to the same trajectory in evolution of their dynamical states (Akhmet & Fen, 2015; Parlitz et al., 1997; Pecora & Carroll, 2015), and fractal entrainment occurring on multiple time scales (Lowen & Teich, 2005; Marmelat, 2014; Rhea et al., 2014). There is significant evidence that auditory nerve firing patterns exhibit such fractal coding, the dimensionality of which can be modulated aperiodically by environmental input, including potentially by speech and music (Lowen & Teich, 2005; Teich, 1989).

There are additional aspects of the strict definition that are worth reconsideration. One is the requirement that the entrainment process outlast, in an oscillatory manner, the end of the entraining stimulus (Obleser & Keyser, 2019). We argue that this may or may not be part of an entrainment mechanism (but is not required). The post-stimulus decay may be near instantaneous, particularly in strongly coupled systems with step-function decay (e.g., electric or laser systems), but also for critically damped (or overdamped) neural systems that do not overshoot (no forward entrainment). Second, the entrainment process need not necessarily be directionally causal (an entraining and entrained system), but rather the coupled systems may be mutually interactive with synchronous activity resulting from bidirectional energy transfer and mode-locking at equilibrium. This interactive aspect of coupled nonlinear oscillators is, in fact, how Huygens originally defined antiphasic entrainment in physical systems.^3^ Note also that the definition of entrainment as an iterative phase-resetting process in endogenous oscillators is a relatively recent development in neuroscience and less frequent in usage than the broader definition of entrainment in physics. In our view, the proposed narrow (strict) definition aims to promote a particular and valuable perspective about the functional significance of periodic cortical oscillations, but is neither sufficiently comprehensive nor universally established in neuroscience or physics. Even Obleser and Keyser (2019) acknowledge that “the more common term ‘synchronization’ could be used instead” of the term entrainment to describe their narrow definition, and Wilsch et al. (2020), noting the limiting nature of the definition, analyze their data in light of a broader and more nuanced perspective, concluding that they have observed lowpass synchronization but have not found “conclusive evidence” for frequency-specific (narrowband) entrainment per the strict definition. From this standpoint, phase resetting is a subtype of physical entrainment that is phenomenologically different than aperiodic, stochastic, or fractal entrainment, which also capture the ongoing nonlinear dynamics of an entrained neural system.

More importantly, and relevant to the current review, the narrow neural definition of entrainment does not naturally extend to psychophysics, where the term is used descriptively to represent a wide-ranging set of phenomena in which performance is temporally correlated with a modulating rhythm. Psychophysical studies of entrained performance do not typically take a position on what the specific underlying neural mechanisms might be (e.g., endogenous oscillators), but rather attempt to model behavioral data on a different scale of analysis, i.e., in the context of potential cognitive or perceptual mechanisms that give rise to the observed patterns of performance (e.g., voluntary selective attention, involuntary attentional capture, ringing of modulation filters, listening in the dip strategy at favorable SNRs, symbolic or cognitive cuing, priming, etc.). The questions addressed by psychophysical studies are therefore often quite different than those probed by neural studies of entrainment, and direct causal inferences should not be drawn without compelling evidence beyond correlative measures.

### Forward entrainment

Forward entrainment describes that part of the entrainment process that outlasts the entraining stimulus. Forward entrainment has been shown using a variety of psychophysical methods (detection, discrimination, and RT designs), with a variety of target signals (tones, noise pulses, temporal gaps, or silent intervals), a variety of entraining stimuli (sinusoidal or square-wave modulated noise, triangular or rectangular tone pulse sequences), in different modalities (auditory, visual, tactile; Jones, 2019), across modalities (audiovisual, auditory-motor^4^; Bouvet et al., 2018), using different neurophysiological techniques (MEG, EEG, ECoG, CSD, and multiunit recordings), and in different species.

How robust is forward entrainment? There are a number of conditions under which forward entrainment fails to be observed. These could potentially be associated with methodological or stimulus design differences. Prior experience, inattention, and intersubject variability may also play a role. Some psychophysical studies that have shown forward entrainment, have also reported the failure of a proportion of their subjects to show the effect under the same experimental conditions (Bauer et al., 2015; Jones et al., 2002; Lawrence et al., 2014). SNR has also been shown to affect the strength of forward entrainment, with weaker or non-existent effects at low or high SNRs. Some studies have shown forward entrainment that lasts for more than one cycle of expected modulation, both behaviorally (de Graaf et al., 2013; Farahbod et al., 2020; Hickok et al., 2015; Jones et al., 2002; Spaak et al., 2014) and neurophysiologically (de Graaf et al., 2013; Lakatos et al. 2013; Spaak et al., 2014), and others have shown an effect that lasts only a single cycle (Barnes & Jones, 2000; Forseth et al., 2020), though the latter have often restricted their measurements to one post-stimulus cycle. Both neural and psychophysical studies have shown that forward entrainment dissipates rapidly and is usually nonexistent (at least behaviorally) by the third or fourth cycle after the end of the entraining stimulus. In our view, while the effect has been demonstrated in a large number of studies, it is sensitive to several factors that are not yet fully understood or explored (Saberi & Hickok, 2022a,b). We have enumerated some of these but additional studies are warranted to understand which factors (positively or negatively) influence the salience of forward entrainment either behaviorally or neurophysiologically.

### Simultaneous versus forward entrainment

Most prior studies have focused on simultaneous entrainment in which the entraining and entrained processes are concurrently active (Henry and Obleser, 2012; ten Oever et al., 2014; Bauer et al., 2018; for reviews see VanRullen et al., 2011 and Haegens and Zion Golumbic, 2018). The current study is the first review paper to exclusively focus on forward entrainment. Simultaneous and forward entrainment are clearly related but distinct phenomena. In simultaneous entrainment, phase effects on detection of target signals are more reliable (smaller variance) and do not typically decay with time since the process is reset at every repetition cycle of the entraining stimulus. In fact, in some cases, there is a build-up (instead of decay) of the entrainment effect (Bauer et al., 2018; van Bree et al., 2021). There are also differences between simultaneous and forward entrainment in measurement of signal predictability. In the latter case, there is no question that entrainment affects processing of future signals locked into a pattern of information change set by the entraining stimulus. This cannot be stated unambiguously in the case of simultaneous entrainment where events to be detected coincide in time with some feature of the ongoing signal. As a consequence, predictive effects cannot be disentangled from ongoing neural processes that potentially include forward and backward masking, comodulation masking release across frequency channels (see below), evoked neural responses, neural inhibition, and a variety of other phenomena that confound interpretation of the entrainment process when the entrained process co-occurs with the entraining stimulus. Furthermore, in simultaneous entrainment paradigms, the entraining pattern need not be fixed but may dynamically vary (as is typical under natural and real-world conditions; see Butzin et al., 2015). The implications for how this affects signal predictability has not been carefully studied. How quickly does the entrained response (neural or behavioral) adapt to new and dynamically changing patterns? Even in the case of fixed modulation rates (and envelope shapes), predictive measurements in simultaneous entrainment are restricted to a single cycle (as contrasted to the sustained activity lasting multiple cycles in forward entrainment). This is because unless the entraining stimulus is dynamically changing in rate or some other physical aspect, one cannot isolate the nonlinear effects of one entrainment cycle from the next in simultaneous entrainment. There may also be differences between forward and simultaneous entrainment in terms of modulation rates to which each may be sensitive. Some evidence suggests that in addition to rate selectivity below ~6 Hz (consistent with forward entrainment; Farahbod et al., 2020) simultaneous entrainment may also be observed at a second higher range of rates between 30 to 40 Hz (Galambos et al., 1981; Teng et al., 2017, Teng and Poeppel, 2020). However, this is likely a categorically different phenomenon and unrelated to the type of attention-driven entrainment discussed here as cognitive processes such as attention (even involuntary attentional capture) cannot sequentially shift between events 40 times per second.

Are simultaneous and forward entrainment in signal detection related to simultaneous and forward *masking*, and how do they potentially relate to energetic versus informational masking? Signal detection in stationary noise is primarily, but not exclusively, limited by energetic masking in the passband of auditory filters centered on the signal frequency (i.e., the critical band; Green and Swets, 1966). This is the type of masking that largely limited detection of signals in the steady state part of noise maskers used by Hickok et al. (2015). In addition to energetic masking, however, information derived from the entraining stimulus as to the expected temporal position of masker dips may also have implicitly directed attention to times at which SNR may have been expected to be most favorable.^5^ What is facilitated, however, isn’t an *informational* contrast (figure-ground) as is typical in studies of informational unmasking that invoke voluntary attention, but rather a process that implicitly captures attention. As such, we do not think that the psychophysical patterns of performance in forward entrainment are directly related to informational (un)masking beyond directed attention. Is forward entrainment related to forward (or backward) masking? The detection of a signal in quiet after termination of a masking noise is affected by several factors, including masker level, temporal separation of masker and signal, masker and signal frequency content, and masker and signal duration. Forward masking could be as large as 40 to 50 dB, decays rapidly and linearly as a function of log delay (between end of masker and onset of signal), but could still be as large as 8 to 10 dB at a delay of 100 ms (Elliott, 1971; Jesteadt et al., 1982). Interestingly, in tone-on-tone forward masking, the phase of the signal relative to the phase of the masker (had the masker continued) affects the amount of forward masking. When the signal is in phase with the masker, forward masking is approximately 3.5 dB larger than when the signal and masker tones are antiphasic (Jesteadt et al., 1982). This parallels the antiphasic effects that we and others have observed behaviorally in forward entrainment. Therefore, there may be some contribution of temporal masking in entrainment.

### Functional and theoretical significance

Forward entrainment may contribute to how the brain encodes complex sounds such as speech and music. Studies have shown that the dynamically changing phase of theta-band oscillations (4–8 Hz) in the brain reliably tracks (i.e., is entrained by) a speech waveform’s envelope, and that the strength of entrainment is correlated with speech intelligibility (Luo & Poeppel, 2007). Since the envelope of speech is not stationary, the instantaneous phase of the quasiperiodic theta-band oscillations resets and “slides” to match the dynamics of the speech waveform. This entrainment process is, in our opinion, not simply passive envelope tracking but has important predictive value. Recent studies have provided evidence that auditory neural responses are shaped by expectations that are hierarchically organized in the cortex (Heilbron & Chait, 2018; Kösem et al., 2018; Okada et al., 2018; van Bree et al., 2021; Zoefell, 2018). These findings are consistent with the theory that the brain constructs a generative model of the world (based on expectations) that informs (and possibly drives) bottom-up processes elicited by external stimuli. The predictive model is recursively updated based on the error that signifies a “bad fit” of the top-down model to the incoming bottom-up signal (Carbajal & Malmierca, 2018; Clark, 2013; Friston, 2012; Heilbron & Chait, 2018). Attention, which we believe plays a key role in forward entrainment, weights the sensory signal in the updated top-down model based on the variance of that signal, with low signal reliability causing a down-weighting of the generated predictive error (i.e., the “bad fit”), and high reliability (low variance) resulting in a prioritization of the error in updating the model (Heilbron & Chait, 2018). Speech envelope tracking by theta-band activity may similarly leverage forward entrainment as a generative model of speech-segment timing to more efficiently process upcoming segments (where timing is inferred from expectations and priors in a Bayesian sense).

Finally, we’d like to conclude by noting that there are several well-established psychophysical phenomena that are possibly related to simultaneous and forward entrainment. This link has not previously been made in the literature. These include co-modulation masking release (CMR; Buss et al., 2012; Hall et al., 1984), co-modulation detection differences (CDD; McFadden, 1987; Verhey & Nitschmann, 2019; Wright, 1990), and modulation detection interference (MDI; Chatterjee & Kulkarni, 2018; Sheft & Yost, 2007; Yost et al., 1989). These processes have been extensively studied in the field of auditory psychophysics and relate to how the modulation pattern in one frequency band affects psychophysical performance in a remote frequency band (several critical bands away) when the modulation envelopes of the band centered on the signal and the spectrally remote band are correlated. It is important to note that conventional theories of signal detection suggest that the detection of a signal (tone) is not affected by noise that is spectrally outside the signal’s critical band. Several across-frequency-channel effects, such as those noted above, violate critical-band predictions for correlated but spectrally distant narrow bands of noise. For example, in CMR, the detection of a signal (tone pulse) in bandlimited noise improves when *additional* noise is presented at a remote frequency band if the two noisebands have correlated envelopes. This process may be interpreted as the capture of the signal-centered noiseband by the remote band, resulting in better isolation of the tonal signal to be detected. CMR has also been observed in a forward-masking paradigm in which the addition of a spectrally remote but correlated noiseband improves the detection of a target tone presented *after* the termination of the masking bands (Wright & McFadden, 1987). In CDD, the detection of a near-threshold narrowband *noise signal* is degraded when a remote noiseband whose envelope is correlated with that of the signal band is presented simultaneously. Detection improves when the bands are uncorrelated. In MDI, the detection of the *modulation* of a suprathreshold noiseband is interfered with by the presence of a remote noiseband with the same modulation rate. What these psychophysical phenomena have in common is that the processing of a signal is improved when the noise that limits its detection is “captured” (or entrained) by spectrally distant noisebands with correlated temporal envelopes.

From an evolutionary standpoint, such cross-channel entrainment has adaptive value. If a predator’s movement generates correlated modulation in spectrally remote bands, it would be advantageous to encode this activity as a single auditory object instead of as multiple sources with separate spectral identities. During ongoing modulation, top-down signals corresponding to that modulation pattern could generate corollary spectrotemporal predictions within each frequency band. Deviations from those predictions could then be augmented or suppressed as adaptive needs dictate to reduce the entropy of the system’s sensory states (Clark, 2013; Friston, 2009; Rao & Ballard, 1999). Forward entrainment may, in this context, instantiate a dynamic auditory afterimage that lasts a fraction of a second to minimize prediction error in signal processing.

## Footnotes

1 Data are reproduced from the original figures using Matlab’s ginput function on figures superimposed on matched axes.

2 We are making two points here, first that many (likely most) local cortical networks are not intrinsically oscillatory and their computations are not characterized by simple sinusoids but rather require much more mathematically complex descriptions, and second because of technical limitations in measurement methodology, their critical role is often missed (or undervalued) in analyses that depend exclusively on extracranial recordings. As the late computational neuroscientist Walter Freeman (2000) notes, the problem is equivalent to observing an internal combustion engine with a stethoscope.

3 Christiaan Huygens invented the pendulum clock to improve maritime navigation. The clock’s pendulum motion allowed robust measurements that could withstand the rigors of sea travel. He conducted tests on pairs of sea clocks for practical redundancy in case one of the clocks stopped or had to be cleaned during travel. Huygens inadvertently physically coupled two clocks by hanging them from the same imperceptibly loose wooden beam and noticed that within half an hour they became synchronized. In a 1665 letter to Sir Robert Moray, read to the Royal Society, Huygens’ discovery is described as “an odd kind of sympathy perceived by him in these watches suspended by the side of each other.” (Bennett et al., 2002). Interestingly, in a letter to his father Huygens notes that while confined at home with a brief illness, he observed that paired clocks always swung 180° degrees out of phase when synchronized. More recent experiments have demonstrated that a small oscillation frequency difference between the two clocks enhances antiphasic synchronization (Czolczynski et al., 2010, 2011; Willms, 2017; Yang et al., 2018). The resultant oscillation frequency of the coupled system is between the oscillation frequencies of the two clocks and does not match one or the other, in contrast to the case of neural or psychophysical studies of entrainment using an external driving modulator that directionally captures the behavior of the entrained system.

4 Most auditory-motor synchronization studies use a finger-tapping procedure to measure simultaneous entrainment to an external rhythmic (often auditory) stimulus. Some studies have extended this work to synchronization-continuation paradigms in which the ability of participants to maintain beat rate is measured after termination of the referent stimulus (Ripp and Su, 2013; McPherson et al., 2018; Ripp & Su, 2013). This work has valuable clinical and therapeutic applications for treatment of several motor dysfunctions (e.g., Parkinson’s disease,; cerebral palsy). The synchronization-continuation paradigm, however, differs from forward entrainment in that it involves a combination of several factors, including voluntary cognitive processes, which allow participants to indefinitely maintain the tapping beat from memory in the absence of the driving stimulus, and as such is different than the brief attentional capture we have described in the current review.

5 What we mean by “attention” here is that the process is not purely a bottom-up effect but has a top-down component with an emphasis on the role of transient attentional capture as contrasted to voluntary attention. It is an open question whether the processes in forward-entrainment can be overridden, for example by explicit instructions to listen at the rhythm defined by the peaks, dips, or a particular feature of the sound sequence (e.g., attending to one tonal frequency in an alternating sequence of two different frequencies). We do know that under some stimulus conditions forward entrainment appears to be overridden, which is one reason why we have concluded that it is attentional (Farahbod et al., 2020).
